# A real-world study of the effectiveness and safety of apatinib-based regimens in metastatic triple-negative breast cancer

**DOI:** 10.1186/s12885-023-11790-6

**Published:** 2024-01-05

**Authors:** Weiwei Huang, Chenxi Wang, Yangkun Shen, Qi Chen, Zhijian Huang, Jian Liu, Xiaoyan Lin, Lili Wang, Fan Wu, Xinhua Chen, Nani Li, Yi Hong, Mulan Chen, Jieyu Li, Chuanzhong Huang

**Affiliations:** 1 Department of Medical Oncology, Fujian Medical University Union Hospital, No.29, Xinquan Road, Gulou District, Fuzhou, Fujian province 350001 China; 2 Department of Medical Oncology, Clinical Oncology School of Fujian Medical University, Fujian Cancer Hospital, No.91, Fuma Road, Jin’an District, Fuzhou, Fujian province 350014 China; 3 Fujian Key Laboratory of Translational Cancer Medicine, Fujian Cancer Hospotial, No.91, Fuma Road, Jin’an District, Fuzhou, Fujian province 350014 China; 4 Fujian Key Laboratory of Innate Immune Biology, Biomedical Research Center of South China, Fujian Normal University Qishan Campus, College Town, Fuzhou, Fujian Province 350117 PR China; 5 Department of Breast Surgical Oncology, Clinical Oncology School of Fujian Medical University, Fujian Cancer Hospital, No. 91, Fuma Road, Jin’an District, Fuzhou, Fujian province 350014 China; 6 Laboratory of Immuno-Oncology, Clinical Oncology School of Fujian Medical University, Fujian Cancer Hospital, No.91, Fuma Road, Jin’an District, Fuzhou, Fujian province 350014 China

**Keywords:** Apatinib, Triple-negative breast cancer, Real-world study

## Abstract

**Purpose:**

This investigation sought to examine the efficacy and safety of low-dose apatinib used alongside chemotherapy in the clinical management of patients with metastatic triple-negative breast cancer (TNBC) within a real-world setting, whilst comparing the outcomes with those treated solely with chemotherapy.

**Methods:**

This case series study analyzed clinical data and treatment outcomes of 163 patients with metastatic TNBC who underwent rescue treatment at the Medical Oncology Department of Clinical Oncology, Fujian Cancer Hospital, School of Fujian Medical University, China, between October 2011 and January 2023. All the patients underwent rescue treatment with either chemotherapy alone or apatinib (250 mg/day) combined with chemotherapy. The study’s primary outcome was progression-free survival (PFS), whereas the secondary outcomes included overall survival (OS), objective response rate (ORR), disease control rate (DCR), and safety profiles.

**Results:**

The study was designed to compare two groups [1]. Out of the 163 TNBC patients who participated in the study, 107 individuals (65.6%) received treatment based on chemotherapy, whereas 56 patients (34.4%) were given treatment based on a combination of low-dose apatinib (250 mg/day) and other treatments, including chemotherapy. After propensity score matching (PSM), the objective response rate (ORR) and disease control rate (DCR) of patients with advanced triple-negative breast cancer (TNBC) who received apatinib-based treatment were 50.0 and 90.0%, respectively, while they were 6.7 and 20.0%, respectively, for the chemotherapy-based group (P < 0.001). The group that received apatinib-based treatment showed superior results in both PFS and OS compared to the group that received chemotherapy. The median PFS and OS for the apatinib-based group were 7.8 and 20.3 months, respectively, while they were only 2.2 months and 9.0 months, respectively, for the chemotherapy-based group (P < 0.001) [2]. Patients who were administered combo therapies, including PD-1 inhibitors, were excluded. In total, 97 patients received chemotherapy alone, while 34 patients were treated with apatinib in combination with chemotherapy. After propensity score matching (PSM), the ORR and DCR for the total group who received combo therapies were 44.4 and 81.5%, respectively, while they were 11.1 and 22.2%, respectively, for the chemotherapy alone group (P < 0.001). The group receiving both apatinib and chemotherapy displayed notable advantages over the group solely receiving chemotherapy in regards to PFS and OS for the entirety of the population. The PFS was found to be 7.8 months in comparison to 2.1 months (P < 0.001) and the OS was 21.1 months in contrast to 9.0 months (P < 0.001). Apatinib combined with chemotherapy induced grade 3/4 hematological toxicities, including neutropenia (8.8%) and thrombocytopenia (2.9%). Additionally, non-hematological toxicities were commonly observed, such as Hand-foot syndrome (35.3%), proteinuria (26.5%), hypertension (61.8%), higher alanine aminotransferase levels (26.5%), and fatigue (35.3%). The most frequent non-hematological grade 3/4 toxicities were Hand-foot syndrome (2.9%) and hypertension (5.9%). The study did not report any fatal adverse effects.

**Conclusions:**

The combination of low-dose apatinib with chemotherapy has proven to be more effective than chemotherapy alone in treating metastatic triple-negative breast cancer (TNBC). Additionally, the occurrence of grade 3/4 non-hematologic toxicities was significantly lower compared to the recommended dose of apatinib.

## Introduction

Breast cancer is the most common cancer among women in China and has been persistently increasing with time in the world [1]. Roughly 15–25% of all breast cancers are categorized “triple-negative breast cancers” (TNBC), which is a subtype of breast cancer that tests negatively for progesterone, estrogen, and “human epidermal growth factor-2” (HER-2) [2]. This subtype is more prevalent in young women and typically comes with a poor prognosis and a high risk of metastasis [3]. Currently, there is no established standard treatment approach strategy for TNBC. While targeted drugs, immune checkpoint inhibitors, and antibody-conjugated drugs are obtainable, chemotherapy continues to be the primary treatment for TNBC [4]. Anthracyclines and taxanes are generally utilized as the first-line treatment, either sequentially or in combination. However, the addition of platinum, capecitabine, or gemcitabine to anthracycline and taxane treatments that have failed has demonstrated unsatisfactory efficacy [5].

Vascular endothelial growth factor(VEGF) binds to its receptor and promotes the growth and spread of tumors [6]. Those with TNBC demonstrate higher levels of VEGF expression compared to those without TNBC. As a result, anti-angiogenic drugs, such as apatinib and bevacizumab, are effective in inhibiting tumor development [7]. The addition of bevacizumab to standard capecitabine or anthracycline/taxane protocols led to an increase in “median progression-free survival” (mPFS) among patients suffering from locally recurrent or metastatic TNBC, with acceptable tolerability, according to the results of phase III clinical study (RIBBON-1) [8]. Evidence points towards increased OS with further research showing that the mPFS in TNBC patients significantly improved with the addition of bevacizumab (6.0 vs. 2.7 months; P < 0.001) [9]. The combination of bevacizumab and chemotherapy also prolonged PFS in 621 TNBC patients in 2010, according to the meta-analysis of three trials (E2100, AVADO, and RIBBON-1), although it did not enhance OS. The approval of bevacizumab for the treatment of breast cancer was revoked by the FDA in 2011 due to safety concerns and cost-effectiveness. Further clinical studies are required to assess the effectiveness and safety of anti-angiogenic treatment.

Apatinib is an orally administered, phosphorylated VEGFR2-targeting tyrosine kinase inhibitor (TKI) of the second generation. According to preclinical studies, apatinib effectively inhibits the growth of solid tumors and leukemia [10]. Its application successfully halted xenograft tumor growth, reversed medication resistance, and prevented the proliferation of tumor stem cells and the production of tumor microspheres. It can also prevent the migration of tumor cells and the formation of human umbilical vein endothelial cell tubes [11].

The mechanism of action of apatinib against malignant tumors is intricate. According to the prevailing perspective, apatinib specifically inhibits the “ATP-binding site” of VEGFR-2 located inside the cell, which subsequently obstructs signal transduction downstream [12]. Furthermore, therapeutic effects can be achieved through blocking and inhibiting the phosphorylation of VEGFR-2(pVEGFR2) and downstream extracellular signal-related kinases, as well as preventing the activation of tyrosine kinases, including PDGFRβ, c-Kit, c-SRC, and Ret, which are associated with tumor development [13]. In this way, tumor angiogenesis can be inhibited through multiple targets and tumor inhibition can be induced by promoting apoptosis in tumor cells.

Aptatinib was approved and authorized by the former “China Food and Drug Administration” in 2014 for treating adenocarcinoma of the gastroesophageal junction or progressive gastric cancer in the third and later lines. Currently, clinical trials are underway to evaluate its efficacy as a targeted anti-tumor angiogenesis drug for treating breast cancer, non-small cell lung cancer, and other tumors. In an investigation of single-agent apatinib for advanced TNBC, 25 patients were included in phase IIa; the median progression-free survival (mPFS) and median overall survival (mOS) were 4.6 and 8.3 months, respectively. Out of the 22 evaluated patients,the partial response (PR) rate was 36.4% and the stable disease (SD) rate was 22.7% [14]. Aptatinib treatment may provide benefits to individuals suffering from advanced TNBC. Nevertheless, administering large doses of the drug is not well-tolerated as it leads to significant proteinuria, high blood pressure, and hand-foot syndrome. Following this, further studies have been conducted to explore the use of low-dose apatinib (250 mg/day) with chemo and immune system checkpoint blockers. This retrospective study aimed to investigate the safety and efficacy of low-dose apatinib in patients with TNBC at the single center of " Medical Oncology Department of Clinical Oncology School of Fujian Medical University, Fujian Cancer Hospital “, in addition to assessing the clinical benefits of combining low-dose apatinib with chemotherapy in comparison to chemotherapy alone. The objective of this retrospective study, conducted in a single center and in compliance with the “Helsinki Declaration and Good Clinical Practise Guidelines” is to evaluate the efficacy and safety of the combined use of apatinib and chemotherapy in treating patients with metastatic or unresectable recurrent TNBC.

## Methods

This study comprised patients with metastatic or unresectable recurrent TNBC who underwent apatinib-based therapy at the Medical Oncology Department of Clinical Oncology School of Fujian Medical University, Fujian Cancer Hospital from October 2011 to January 2023. Some patients included in the retrospective data received simultaneous chemotherapy and apatinib as a part of a clinical study initiated by the researchers. Patients compared between groups receiving apatinib-based therapy versus chemo-based therapy, or apatinib plus Chemotherapy versus chemotherapy alone, were matched for age, ECOG PS, menopausal status, and metastasis site or locations. These patients received written informed consent and approval from the institutional ethics committee. Only patients between the ages of 18 and 70 were included. HER2/neu-negative was defined as either immunohistochemistry (IHC) 0–1 + or IHC 2 + fluorescence in situ hybridization (FISH)-negative, while TNBC (ER/PR-negative) was defined as ER/PR staining less than 1%. According to the Response Evaluation Criteria in Solid Tumors (RECIST v1.1), all patients included in the study had detectable lesions and were administered oral apatinib at a dosage of 250 mg/day for a minimum of 30 days. The patient ceased apatinib-based therapy upon refusal, worsening of symptoms, or intolerable side effects. The study utilized RECIST v1.1 to assess efficacy and NCI-CTCAE 5.0 to report adverse events. The primary outcomes centered on PFS. The secondary outcomes considered were the OS, ORR, DCR, and safety profiles. To reduce the risk of selection bias and other confounding factors, propensity score matching (PSM) was utilized. The PSM model included the following factors: patient age, Eastern Co-operative Oncology Group Performance Score (ECOG PS), menopausal status, prior surgery, TNBC at initial onset, Ki67 status, metastasis site or locations, perioperative treatment, combination therapy type and line of therapy. Matched pairs were then formed using a 1-to-1 nearest-neighbor with a caliper width of 0.2. Between-group differences were compared using a Student’s t-test or the chi-squared test. OS and PFS were calculated with the Kaplan–Meier method and compared using the log-rank test. Any factors that were statistically significant (p < 0.10) in the univariate analysis were candidates for entry into a multivariable Cox proportional-hazards model. All p-values were 2-sided, with p-values < 0.05 considered significant. In this study, the prognostic model was built to assess the contribution of variables, which was formed from a training set of 70% (114) and a test set of the remaining 30% (49) randomly, derived from the original data. Shapley additive explanations (SHAP) were used to explain the model. “survival”, “shapviz” and “xgboost” R packages were used to perform the SHAP analysis. R version 4.2.3 was used for all statistical analyses.

## Results

Initially, 273 patients who consented to the rescue treatment plan underwent screening. Following the elimination of individuals with missing data or lost follow-up, 163 eligible patients with TNBC were selected for investigation. Of these, 56 patients (34.4%) underwent apatinib-based treatment, while the remaining 107 patients (65.6%) received chemotherapy-based rescue treatment. Thirty-four patients were administered apatinib in combination with chemotherapy, excluding those who received combination treatment containing PD-1 inhibitors (18 cases of apatinib + PD-1 inhibitor + chemotherapy), apatinib monotherapy (2 cases), apatinib with PD-1 inhibitor (1 case), and apatinib with PARP inhibitor (1 case). Ninety-seven patients received chemotherapy alone, except for four cases of chemotherapy with a PD-1 inhibitor, one case of chemotherapy with HER2-targeted therapy, three cases of Chemotherapy combined with endocrine therapy containing CDK4/6 inhibitors, one case of chemotherapy with bevacizumab, and one case of chemotherapy with PARP inhibitor.

**Fig. 1:**
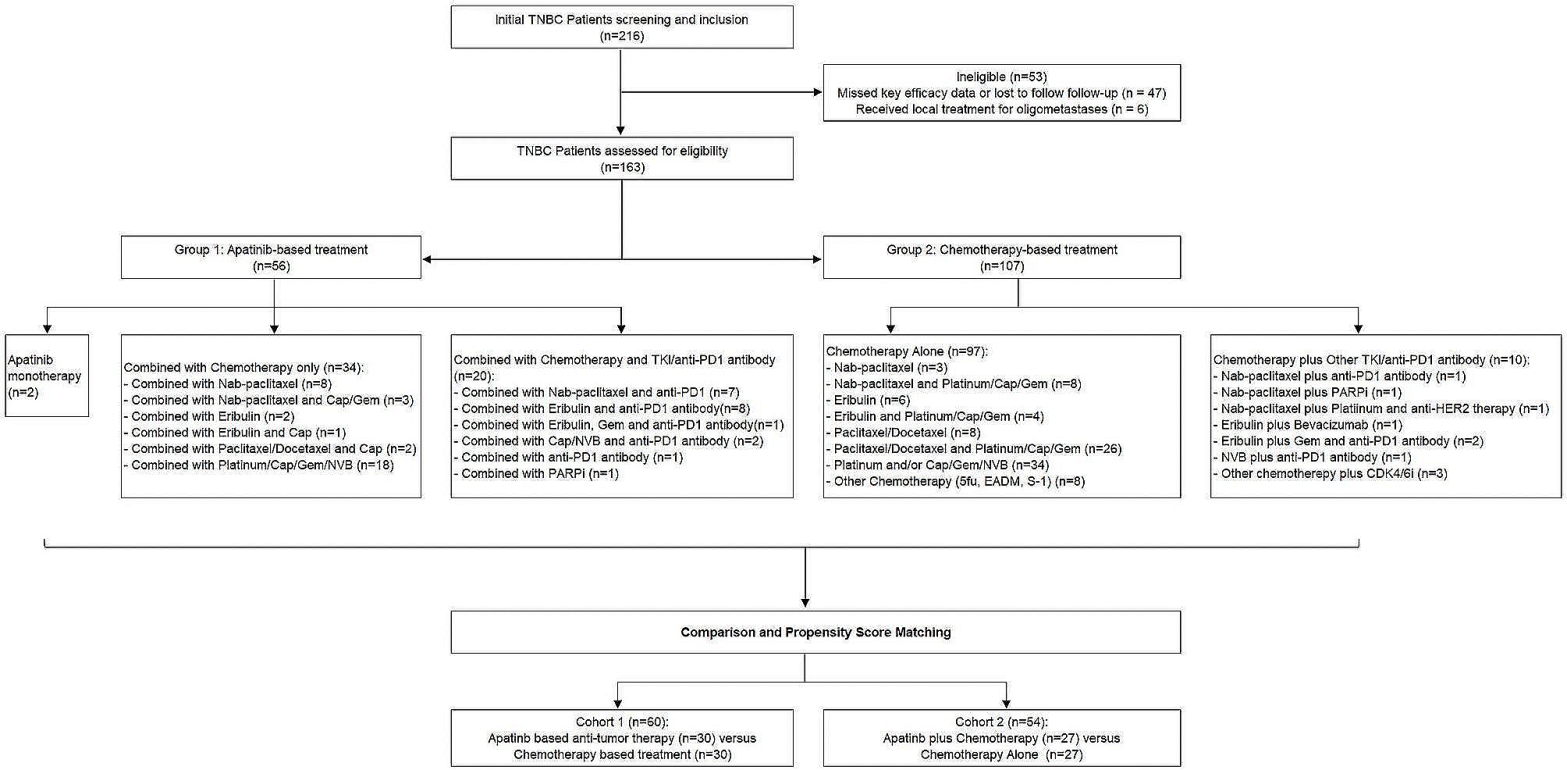
Population attrition after applying inclusion/exclusion criteria and propensity score matching. Cap, capecitabine; Gem, gemcitabine; NVB, vinorelbine; 5fu, 5-fluorouracil; EADM, epirubicin

The study was designed to compare two groups: (1) a group that received apatinib (n = 56) versus a group that did not (n = 107); (2) a group that received apatinib in combination with chemotherapy (n = 34) versus a group that received only chemotherapy (n = 97). After PSM, the two comparison cohorts were as follows; apatinib-based treatment (n = 30) versus chemotherapy-based treatment (n = 30), apatinib combined with chemotherapy treatment (n = 27) versus chemotherapy alone treatment (n = 27). The detailed information regarding treatment is shown in Fig. 1.

**Fig. 2:**
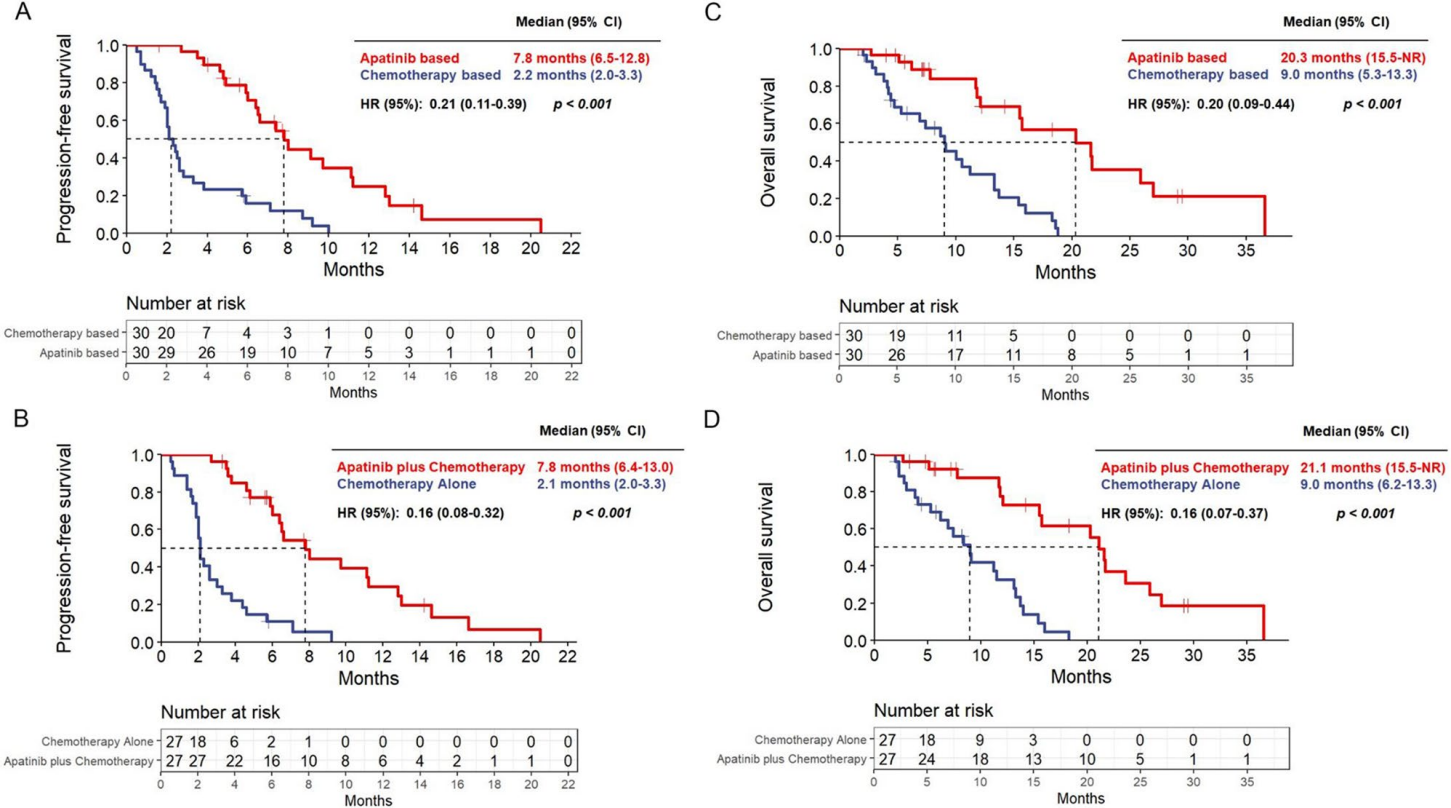
Progression-free survival (**A**) and overall survival outcomes. Kaplan-Meier analysis of survival showing progression-free survival (**A**) and overall survival (**C**) in cohort 1, and progression-free survival (**B**) and overall survival (**D**) in cohort 2. Kaplan-Meier estimates of progression-free survival by Response Evaluation Criteria in Solid Tumors version 1.1. HR = hazard ratio

**Fig. 3:**
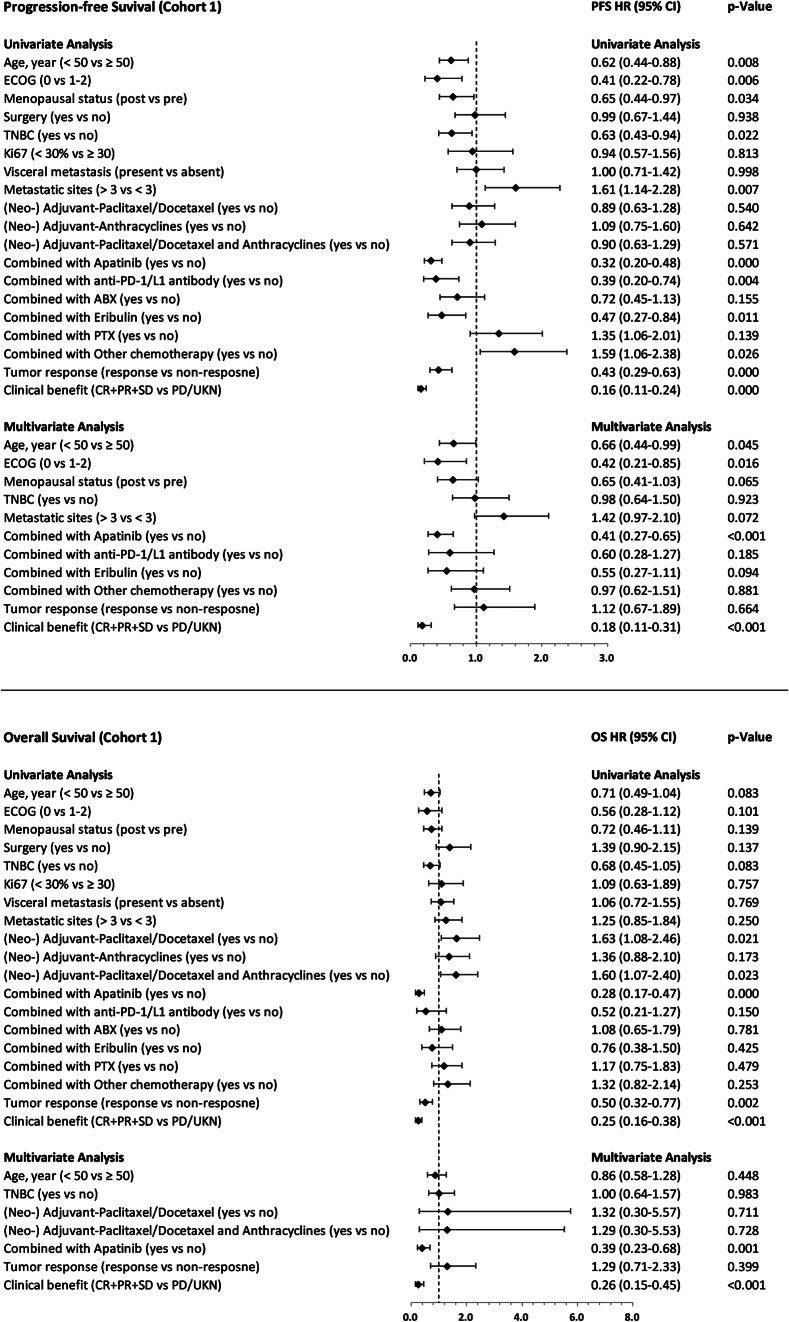
Forest plots. Showing the association between risk factors and progression-free survival or overall survival in cohort 1. (Cox regression, HR, and 95% CI). P value was calculated with a 2-sided log-rank test. Any factors that were statistically significant at P < 0.1 in the univariate analysis were candidates for entry into a multivariable Cox analysis

**Fig. 4:**
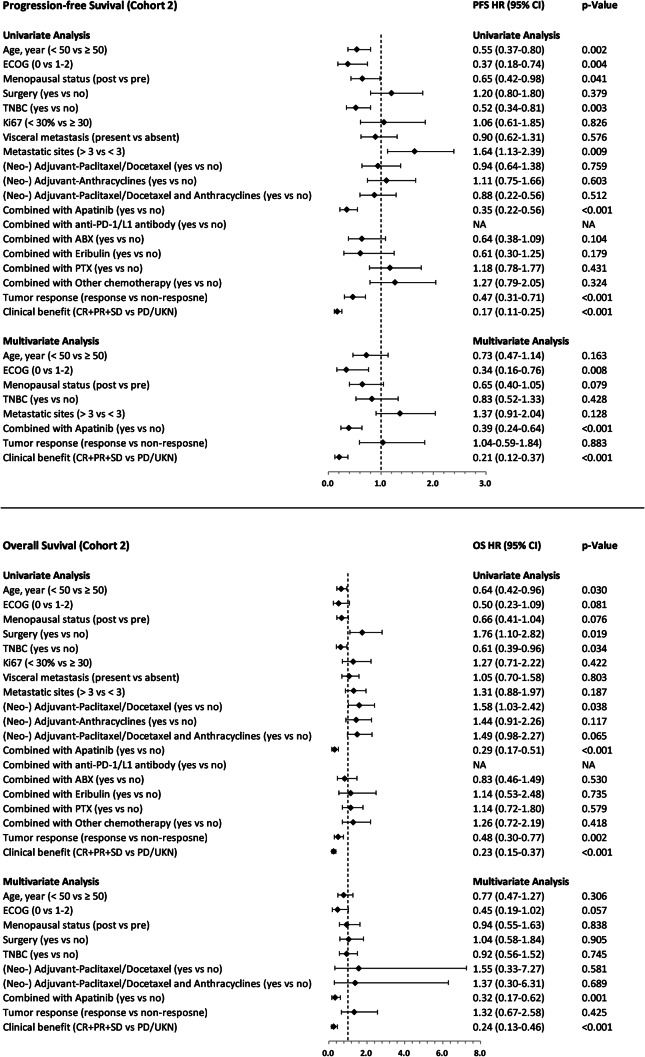
Forest plots. Showing the association between risk factors and progression-free survival or overall survival in cohort 2. (Cox regression, HR, and 95% CI). P value was calculated with a 2-sided log-rank test. Any factors that were statistically significant at P < 0.1 in the univariate analysis were candidates for entry into a multivariable Cox analysis

**Fig. 5:**
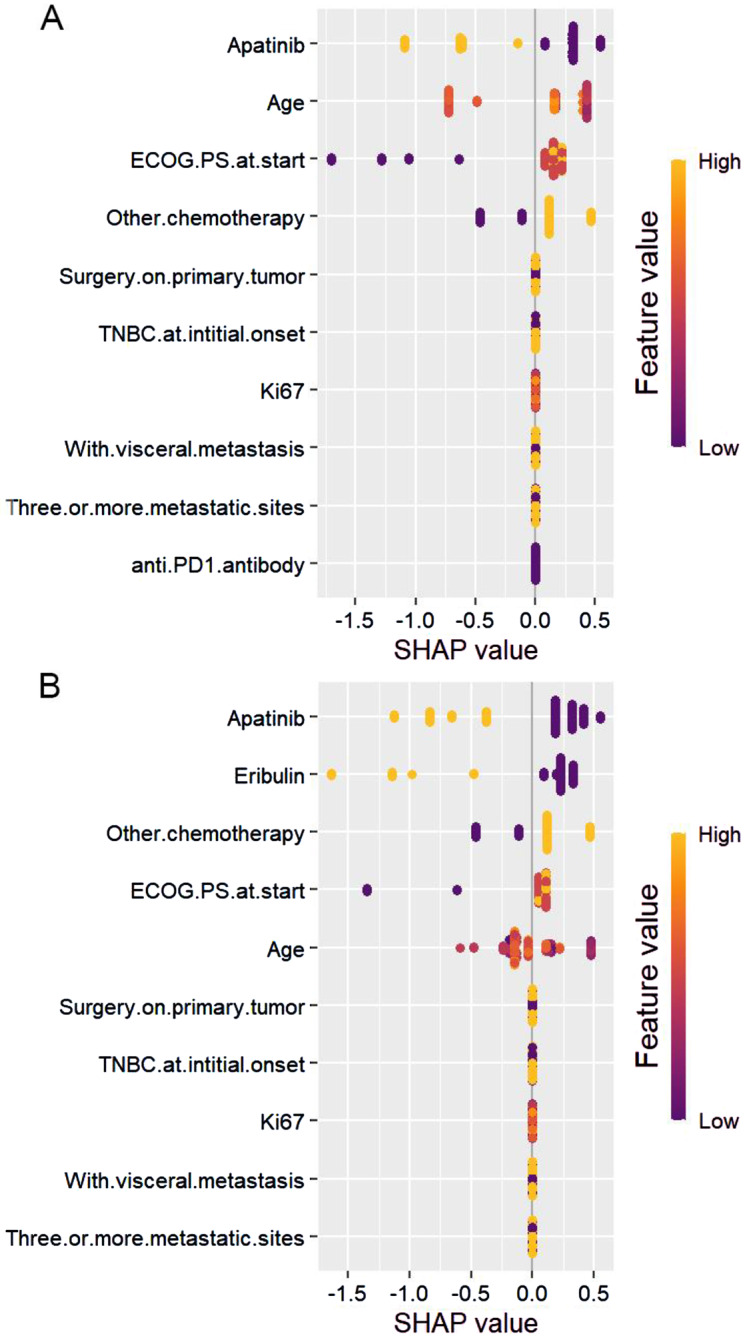
Summary plots for SHAP values. (**A**) Progression-free survival. (**B**) Overall survival. For each predictor, one point corresponds to a single patient, and the x-axis represents the impact of the feature on the model’s output for the specific patient. A positive SHAP value contributes to disease progression or death, while a negative value contributes to OS or PFS. Predictors are arranged along the y-axis based on their ranking: the higher the feature is positioned in the plot, the more significant it is in the model

Baseline characteristics and disease characteristics pre- and post-PSM for the apatinib-based treatment versus chemotherapy-based treatment, apatinib combined with chemotherapy treatment versus chemotherapy alone treatment are shown in Tables 1 and 2, respectively. Table 3 and Table 4, as well as Figs. 2, 3, 4 and 5, illustrate the efficacy data.

**Table 1:** Patient baseline demographic and clinical characteristics in cohort 1 (Apatinib-based vs. Chemotherapy-based)

	Initial cohort				Propensity-score-matched cohort				
	Apatinib Based (n = 56)	Chemotherapy Based (n = 107)	*P*			Apatinib Based (n = 30)	Chemotherapy Based (n = 30)	*P*	
Age, years									
< 50	26 (46.4)	54 (50.5)	*0.745*			16 (53.3)	18 (60.0)	*0.794*	
≥ 50	30 (53.6)	53 (49.5)				14 (46.7)	12 (40.0)		
ECOG PS at start									
0	7 (12.5)	13 (12.1)	*1.000*			3 (10.0)	2 (6.7)	*1.000*	
≥ 1	49 (87.5)	94 (87.9)				27 (90.0)	28 (93.3)		
Menopausal status at diagnosis									
Premenopausal	41 (73.2)	80 (74.8)	*0.979*			7 (23.3)	6 (20.0)	*1.000*	
Postmenopausal	15 (26.8)	27 (25.2)				23 (76.7)	24 (80.0)		
Surgery on primary tumor									
Yes	37 (66.1)	83 (77.6)	*0.163*			21 (70.0)	26 (86.7)	*0.210*	
No	19 (33.9)	24 (22.4)				9 (30.0)	4 (13.3)		
TNBC at the initial onset									
Yes	48 (85.7)	76 (71.0)	*0.058*			26 (86.7)	23 (76.7)	*0.505*	
No	8 (14.3)	31 (29.0)				4 (13.3)	7 (23.3)		
Ki67 ≥ 30%	48 (85.7)	94 (87.9)	*0.888*			27 (90.0)	28 (93.3)	*1.000*	
Metastatic sites									
Visceral	29 (51.8)	59 (55.1)	*0.808*			15 (50.0)	17 (56.7)	*0.796*	
Non-visceral	27 (48.2)	48 (44.9)				15 (50.0)	13 (43.3)		
Metastatic sites > 3	16 (28.6)	54 (50.5)	*0.012*			9 (30.0)	12 (40.0)	*0.588*	
Location of metastases									
Brain	2 (3.6)	7 (6.5)	*0.669*			1 (3.3)	0 (0.0)	*1.000*	
Bone	19 (33.9)	49 (45.8)	*0.196*			9 (30.0)	9 (30.0)	*1.000*	
Liver	8 (14.3)	23 (21.5)	*0.366*			4 (13.3)	5 (16.7)	*1.000*	
Lung	22 (39.3)	45 (42.1)	*0.862*			11 (36.7)	13 (43.3)	*0.792*	
Lymph node	40 (71.4)	76 (71.0)	*1.000*			21 (70.0)	22 (73.3)	*1.000*	
Adrenal glands	1 (1.8)	3 (2.8)	*1.000*			1 (3.3)	1 (3.3)	*1.000*	
Chest wall	5 (8.9)	15 (14.0)	*0.491*			3 (10.0)	3 (10.0)	*1.000*	
(Neo-) Adjuvant therapies									
Paclitaxel/Docetaxel	34 (60.7)	73 (68.2)	*0.432*			19 (63.3)	20 (66.7)	*1.000*	
Anthracyclines	33 (58.9)	81 (75.7)	*0.042*			19 (63.3)	20 (66.7)	*1.000*	
Paclitaxel/Docetaxel and Anthracyclines	31 (55.4)	71 (66.4)	*0.227*			18 (60.0)	20 (66.7)	*0.789*	
Platinum (Cis/Carbo)	4 (7.1)	4 (3.7)	*0.566*			2 (6.7)	2 (6.7)	*1.000*	
Capecitabine	4 (7.1)	4 (3.7)	*0.566*			2 (6.7)	2 (6.7)	*1.000*	
Treatment									
anti-PD-1/L1 antibody	19 (33.9)	4 (3.7)	*< 0.001*			5 (16.7)	3 (10.0)	*0.704*	
Nab-paclitaxel	18 (32.1)	14 (13.1)	*0.007*			6 (20.0)	5 (16.7)	*1.000*	
Eribulin	12 (21.4)	13 (12.1)	*0.183*			6 (20.0)	3 (10.0)	*0.470*	
Paclitaxel/Docetaxel	2 (3.6)	34 (31.8)	*< 0.001*			2 (6.7)	3 (10.0)	*1.000*	
Other chemotherapy (Platinum, Capecitabine, Gemcitabine, NVB, etc.)	27 (48.2)	87 (81.3)	*< 0.001*			21 (70.0)	24 (80.0)	*0.551*	
Lines of therapy									
1st Line	16 (28.6)	59 (55.1)	*0.002*			8 (26.7)	8 (26.7)	*1.000*	
2^nd+^ Line	40 (71.4)	48 (44.9)				22 (73.3)	22 (73.3)		

**Table 2:** Patient baseline demographic and clinical characteristics in cohort 2 (Apatinib plus Chemotherapy vs. Chemotherapy Alone)

	Initial cohort				Propensity-score-matched cohort				
	Apatinib plus Chemotherapy (n = 34)	Chemotherapy Alone (n = 97)	*P*			Apatinib plus Chemotherapy (n = 27)	Chemotherapy Alone (n = 27)	*P*	
Age, years									
< 50	15 (44.1)	49 (50.5)	*0.658*			13 (48.1)	16 (59.3)	*0.585*	
≥ 50	19 (55.9)	48 (49.5)				14 (51.9)	11 (40.7)		
ECOG PS at start									
0	5 (14.7)	11 (11.3)	*0.833*			3 (11.1)	1 (3.7)	*0.603*	
≥ 1	29 (85.3)	86 (88.7)				24 (88.9)	26 (96.3)		
Menopausal status at diagnosis									
Premenopausal	23 (67.6)	71 (73.2)	*0.691*			19 (70.4)	20 (74.1)	*1.000*	
Postmenopausal	11 (32.4)	26 (26.8)				8 (29.6)	7 (25.9)		
Surgery on primary tumor									
Yes	20 (58.8)	74 (76.3)	*0.084*			17 (63.0)	22 (81.5)	*0.224*	
No	14 (41.2)	23 (23.7)				10 (37.0)	5 (18.5)		
TNBC at the initial onset									
Yes	30 (88.2)	70 (72.2)	*0.096*			24 (88.9)	24 (88.9)	*1.000*	
No	4 (11.8)	27 (27.8)				3 (11.1)	3 (11.1)		
Ki67 ≥ 30%	29 (85.3)	85 (87.6)	*0.958*			23 (85.2)	25 (92.6)	*0.665*	
Metastatic sites									
Visceral	19 (55.9)	53 (54.6)	*1.000*			13 (48.1)	15 (55.6)	*0.785*	
Non-visceral	15 (44.1)	44 (45.4)				14 (51.9)	12 (44.4)		
Metastatic sites > 3	9 (26.5)	50 (51.5)	*0.020*			8 (29.6)	10 (37.0)	*0.773*	
Location of metastases									
Brain	1 (2.9)	6 (6.2)	*0.779*			0	0	*NA*	
Bone	12 (35.3)	45 (46.4)	*0.356*			8 (29.6)	8 (29.6)	*1.000*	
Liver	5 (14.7)	19 (19.6)	*0.707*			4 (14.8)	4 (14.8)	*1.000*	
Lung	15 (44.1)	41 (42.3)	*1.000*			11 (40.7)	13 (48.1)	*0.784*	
Lymph node	25 (73.5)	70 (72.2)	*1.000*			19 (70.4)	20 (74.1)	*1.000*	
Adrenal glands	0	2 (2.1)	*0.975*			0	0	*NA*	
Chest wall	3 (8.8)	13 (13.4)	*0.691*			3 (11.1)	4 (14.8)	*1.000*	
(Neo-) Adjuvant therapies									
Paclitaxel/Docetaxel	20 (58.8)	63 (64.9)	*0.666*			17 (63.0)	18 (66.7)	*1.000*	
Anthracyclines	20 (58.8)	71 (73.2)	*0.177*			17 (63.0)	18 (66.7)	*1.000*	
Paclitaxel/Docetaxel and Anthracyclines	20 (58.8)	61 (62.9)	*0.830*			17 (63.0)	18 (66.7)	*1.000*	
Platinum (Cis/Carbo)	2 (5.9)	3 (3.1)	*0.833*			2 (7.4)	1 (3.7)	*1.000*	
Capecitabine	2 (5.9)	3 (3.1)	*0.833*			2 (7.4)	1 (3.7)	*1.000*	
Treatment									
anti-PD-1/L1 antibody	0	0	*NA*			0	0	*NA*	
Nab-paclitaxel	11 (32.4)	11 (11.3)	*0.011*			6 (22.2)	5 (18.5)	*1.000*	
Eribulin	3 (8.8)	10 (10.3)	*1.000*			2 (7.4)	2 (7.4)	*1.000*	
Paclitaxel/Docetaxel	2 (5.9)	34 (35.1)	*0.002*			2 (7.4)	1 (3.7)	*1.000*	
Other chemotherapy (Platinum, Capecitabine, Gemcitabine, NVB, etc.)	24 (70.6)	80 (82.5)	*0.219*			22 (81.5)	25 (92.6)	*0.418*	
Lines of therapy									
1st Line	6 (17.6)	53 (54.6)	*< 0.001*			6 (22.2)	6 (22.2)	*1.000*	
2^nd+^ Line	28 (82.4)	44 (45.4)				21 (77.8)	21 (77.8)		

**Table 3:** Summary of efficacy after PSM in cohort 1 (Apatinib-based vs. Chemotherapy-based)

	Apatinib based	Chemotherapy based	hazard ratio	*P*
**All comers, n**	30	30	-	*-*
Objective response, n (%; 95% CI)	15 (50.0; 31.3–68.7)	2 (6.7; 0.8–22.1)		*< 0.001*
Disease control, n (%; 95% CI)	27 (90.0; 73.5–97.9)	6 (20; 7.7–38.6)		*< 0.001*
Progression-free survival, month (95% CI)	7.8 (6.5–12.8)	2.2 (2.0-3.3)	0.21 (0.11–0.39)	*< 0.001*
Overall survival, month (95% CI)	20.3 (15.5-NR)	9.0 (5.3–13.3)	0.20 (0.09–0.44)	*< 0.001*
**1st line, n**	8	8	-	*-*
Objective response, n (%; 95% CI)	7 (87.5; 47.3–99.7))	1 (12.5; 0.3–52.7)	-	*0.010*
Disease control, n (%; 95% CI)	8 (100.0)	2 (25.0; 3.2–65.1)	-	*0.007*
**2** ^**nd+**^ **line, n**	22	22	-	*-*
Objective response, n (%; 95% CI)	8 (36.4; 17.2–59.3)	1 (4.5; 0.1–22.8)	-	*0.025*
Disease control, n (%; 95% CI)	18 (81.8; 59.7–94.8)	4 (18.2; 5.2–40.3)	-	*< 0.001*

**Table 4:** Summary of efficacy after PSM in cohort 2 (Apatinib plus Chemotherapy vs. Chemotherapy Alone)

	Apatinib plus Chemotherapy	Chemotherapy alone	hazard ratio	*P*
**All comers, n**	27	27	-	*-*
Objective response, n (%; 95% CI)	12 (44.4; 25.5–64.7)	3 (11.1; 2.4–29.2)		*0.015*
Disease control, n (%; 95% CI)	22 (81.5; 61.9–93.7)	6 (22.2; 8.6–42.5)		*< 0.001*
Progression-free survival, month (95% CI)	7.8 (6.4–13.0)	2.1 (2.0-3.3)	0.16 (0.08–0.32)	*< 0.001*
Overall survival, month (95% CI)	21.1 (15.5-NR)	9.0 (6.2–13.3)	0.16 (0.07–0.37)	*< 0.001*
**1st line, n**	6	6		*-*
Objective response, n (%; 95% CI)	5 (83.3; 35.9–99.6)	2 (33.3; 4.3–77.7)		*0.242*
Disease control, n (%; 95% CI)	6 (100.0)	2 (33.3; 4.3–77.7)		*0.030*
**2** ^**nd+**^ **line, n**	21	21		*-*
Objective response, n (%; 95% CI)	7 (33.3; 14.6–57.0)	1 (4.8; 0.1–23.8)		*0.049*
Disease control, n (%; 95% CI)	16 (76.2; 52.8–91.8)	4 (19.0; 5.4–41.9)		*0.001*

### Effectiveness evaluation

#### Apatinib-based versus chemotherapy-based therapy

After PSM, the group receiving apatinib-based(n = 30) was compared with the group receiving chemotherapy-based rescue treatment (n = 30). Results showed that after receiving apatinib, statistically significant improvements in ORR and DCR were observed in the overall population, as well as in the first-line and second-and later-line advanced TNBC populations (Table 3). In addition, The PFS of the group receiving apatinib-based treatment showed a substantial improvement (7.8 vs. 2.2 months; HR = 0.21, p < 0.001) (Fig. 2A). The OS of the entire population was significantly improved (20.3 vs. 9.0 months; HR = 0.20, p < 0.001) (Fig. 2C).

The results of the univariate and multivariate analyses for PFS and OS are shown in Fig. 3. The results showed that the age (less than 50 vs. greater than or equal to 50, HR = 0.66; 95% CI: 0.44–0.99, p = 0.045), ECOG score (0 vs. 1–2, HR = 0.42; 95% CI: 0.21–0.85, p = 0.016), combination therapy with apatinib (HR = 0.41; 95% CI: 0.27–0.65, p < 0.001) and clinical benefit profile (CR + PR + SD vs. PD/UKN, HR = 0.18; 95% CI: 0.11–0.31, p < 0.001) were independent predictors of longer PFS. In addition, combination therapy with apatinib (HR = 0.39; 95% CI: 0.23–0.68, p = 0.001) and clinical benefit profile (CR + PR + SD vs. PD/UKN, HR = 0.26; 95% CI: 0.15–0.45, p < 0.001) were independent predictors for longer OS.

#### Apatinib combined with chemotherapy versus chemotherapy alone therapy

After PSM, the group receiving apatinib combined with chemotherapy (n = 27) was compared with the group receiving chemotherapy alone rescue treatment (n = 27). The study showed that receiving a combination of chemotherapy and apatinib resulted in a statistically significant improvement in DCR in all populations studied, including the first-line and second- and later-line advanced TNBC populations, as well as a significant difference in ORR in the overall, second-line, and above populations. However, no significant difference in ORR was observed in the first-line population (Table 4). In addition, The PFS of the group receiving apatinib combined with chemotherapy treatment showed a substantial improvement (7.8 vs. 2.1 months; HR = 0.16, p < 0.001) (Fig. 2B). The OS of the entire population was significantly improved (21.1 vs. 9.0 months; HR = 0.16, p < 0.001) (Fig. 2D).

The results of the univariate and multivariate analyses for PFS and OS are shown in Fig. 4. The results showed that the ECOG score (0 vs. 1–2, HR = 0.34; 95% CI: 0.16–0.76, p = 0.008), combination therapy with apatinib (HR = 0.39; 95% CI: 0.24–0.64, p < 0.001) and clinical benefit profile (CR + PR + SD vs. PD/UKN, HR = 0.21; 95% CI: 0.12–0.37, p < 0.001) were independent predictors of longer PFS. In addition, combination therapy with apatinib (HR = 0.32; 95% CI: 0.17–0.62, p = 0.001) and clinical benefit profile (CR + PR + SD vs. PD/UKN, HR = 0.24; 95% CI: 0.13–0.46, p < 0.001) were independent predictors for longer OS.

### Feature importance

The summary plots of the SHAP values for the top 10 most significant predictors of PFS and OS are shown in Fig. 5A and B, respectively. The apatinib treatment was considered the most significant variable both in predicting PFS and OS (patients treated with apatinib had lower risk scores, while those who were not received apatinib had a higher risk score), followed by age, ECOG PS and other chemotherapy in predicting PFS, and eribulin, other chemotherapy, ECOG PS and age in predicting OS (Fig. 5).

### Adverse reactions

Grade 3/4 hematological side effects, such as thrombocytopenia (5.4%) and leukopenia (12.5%), were observed in the apatinib-containing group during this trial. Additionally, PD-1 inhibitors and other therapies were administered. The non-hematological toxicities that were common included hand-foot syndrome (39.3%), proteinuria (23.2%), hypertension (58.9%), elevated ALT levels (33.9%), and fatigue (48.2%). Of these, hand-foot syndrome (1.8%), proteinuria (0%), hypertension (5.4%), elevated ALT levels (3.6%), and fatigue (1.8%) were identified as grade 3/4 non-hematological toxicities.

Thrombocytopenia (2.9%) and leukopenia (8.8%) were observed as the grade 3/4 hematological toxicities within the apatinib coupled with the chemotherapy group without PD-1 inhibitor. Hand-foot syndrome (35.3%), proteinuria (26.5%), hypertension (61.8%), elevated ALT levels (26.5%), and fatigue (35.3%) were identified as prevalent non-hematological toxicities. Notably, hand-foot syndrome (2.9%) and hypertension (5.9%) were categorized as grade 3/4 non-hematological toxicities, while no instances of severe proteinuria, elevated ALT levels, or fatigue were observed (refer to Tables 5 and 6).

**Table 5:** Most common adverse events (any grade)

Any Grade, n (%)	Apatinib Based N = 56	Chemotherapy Based N = 107	*P*		Apatinib plus Chemotherapy N = 34	Chemotherapy Alone N = 97	*P*
**Hematological events**							
Neutropenia	45 (80.4)	88 (82.2)	*0.77*		26 (76.5)	79 (81.4)	*0.53*
Anemia	18 (32.1)	57 (53.3)	*0.01*		8 (23.5)	53 (54.6)	*0.002*
Thrombocytopenia	10 (17.9)	17 (15.9)	*0.75*		7 (20.6)	17 (17.5)	*0.69*
**Non-hematological events**							
Peripheral neuropathy	15 (26.8)	51 (47.7)	*0.01*		8 (23.5)	45 (46.4)	*0.02*
Nausea/vomiting	8 (14.3)	39 (36.4)	*0.003*		4 (11.8)	38 (39.2)	*0.006*
Diarrhea	2 (3.6)	16 (15.0)	*0.05*		0	16 (16.5)	*0.01*
AST/ALT alterations	19 (33.9)	36 (33.6)	*0.97*		9 (26.5)	32 (33.0)	*0.48*
Fatigue	27 (48.2)	46 (43.0)	*0.52*		12 (35.3)	39 (40.2)	*0.61*
Proteinuria	13 (23.2)	3 (2.8)	*< 0.001*		9 (26.5)	3 (3.1)	*< 0.001*
Hypertension	33 (58.9)	2 (1.9)	*< 0.001*		21 (61.8)	1 (1.0)	*< 0.001*
Hand-foot Syndrome	22 (39.3)	8 (7.5)	*< 0.001*		12 (35.3)	8 (8.2)	*< 0.001*
Alopecia	29 (51.8)	78 (72.9)	*0.007*		17 (50.0)	72 (74.2)	*0.009*

**Table 6:** Grade 3 to 4 adverse events

Grade 3 to 4, n (%)	Apatinib Based N = 56	Chemotherapy Based N = 107	*P*		Apatinib plus Chemotherapy N = 34	Chemotherapy Alone N = 97	*P*
**Hematological events**							
Neutropenia	7 (12.5)	28 (26.2)	*0.04*		3 (8.8)	26 (26.8)	*0.05*
Anemia	0	6 (5.6)	*0.09*		0	5 (5.2)	*0.33*
Thrombocytopenia	3 (5.4)	4 (3.7)	*0.94*		1 (2.9)	4 (4.1)	*1.00*
**Non-hematological events**							
Peripheral neuropathy	0	1 (0.9)	*1.00*		0	1 (1.0)	*1.00*
Nausea/vomiting	0	3 (2.8)	*0.55*		0	2 (2.1)	*1.00*
Diarrhea	0	0	*-*		0	0	*-*
AST/ALT alterations	2 (3.6)	6 (5.6)	*0.85*		0	6 (6.2)	*0.34*
Fatigue	1 (1.8)	3 (2.8)	*1.00*		0	0	*-*
Proteinuria	0	0	*-*		0	0	*-*
Hypertension	3 (5.4)	0	*0.04*		2 (5.9)	0	*0.07*
Hand-foot Syndrome	1 (1.8)	0	*0.34*		1 (2.9)	0	*0.26*
Alopecia	0	1 (0.9)	*1.00*		0	1 (1.0)	*1.00*

## Discussion

The combination of chemotherapeutic agents and apatinib is an emerging area of research for the treatment of advanced TNBC. There is already evidence of a substantial potential therapeutic effect, but this evidence is not yet comprehensive and adequate. The prescribed apatinib doses for phases IIa and IIb in clinical trial for metastatic TNBC following multiline treatment was 750 mg/day and 500 mg/day, correspondingly [14]. The ORR and DCR for the 56 patients who could be evaluated during the phase IIb trial were 10.7% and 25.0%, respectively. The PFS and OS were 3.3 (95% CI 1.7-5.0) and 10.6 (95% CI 5.6–15.7) months. In the phase IIa trial, the ORR and DCR for the 22 patients who were evaluable were 36.4% and 59.1%, respectively. The median PFS and OS were 4.6 (95% CI 2.1–7.1) and 8.3 (95% CI 4.1–12.4) months, respectively. In the phase IIa trial, the most frequent grade 3/4 hematologic toxicities were thrombocytopenia (8.0%), leukopenia (8.0%), granulocytopenia (4.0%), and anemia (4.0%). The most prevalent grade 3/4 non-hematologic effects were hand-foot syndrome (24.0%), proteinuria (4.0%), hypertension (36.0%), increased ALT (4.0%), and tiredness (8.0%). The recommended dose of apatinib is 500 mg daily, based on safety and efficacy [14]. A trial conducted on advanced non-TNBC patients after multiline treatment, using apatinib 500 mg/day, resulted in an ORR of 16.7% (6/36), and median PFS and OS of 4.0 and 10.3 months, respectively. Out of the total 38 participants in the study, one exhibited complete remission while five had partial remission. Proteinuria (5.1%), hand-foot syndrome (10.3%), and hypertension (20.5%) were the most frequent grade 3/4 treatment-related toxicities [15]. Another retrospective study used capecitabine and 500 mg/day of apatinib as the third-line therapy for metastatic TNBC. When capecitabine was administered alone, the ORR was 13.4% and the DCR was 31.8% among 22 patients. However, when the same 22 patients were treated with combination treatment, the ORR was 40.9% and the DCR was 68.2%, indicating better outcomes than with capecitabine alone (P = 0 0.042;0 0.016). The mean PFS for the combined therapy group was 5.5 months, compared to 3.5 months for the capecitabine group, indicating a notable advantage (P = 0.001). There was no significant difference in the adverse effects of hematological toxicity (reduced white blood cell and granulocyte counts) and non-hematological toxicity (hypertension, fatigue, hand-foot syndrome, vomiting). The combination of apatinib and capecitabine may result in greater efficacy and comparable side effects compared to the capecitabine regimen [16].

Current published regimens of apatinib monotherapy or combination therapy have exhibited efficacy and safety, when compared to other TKI anti-angiogenic drugs like sunitinib and sorafenib, that have displayed inadequate efficacy as monotherapy. Nevertheless, the recommended 500 mg/day dose of apatinib can cause side effects, including hand-foot syndrome, hypertension, and proteinuria, which could hinder future studies. In this study, we investigated the efficacy of administering a low dosage of apatinib (250 mg/day) in combination with chemotherapy for the treatment of advanced TNBC, which had proven resistant to previous therapies. Additionally, we aimed to investigate whether this therapy could decrease the incidence of hypertension, proteinuria, and hand-foot syndrome, and concurrently lead to advancements in PFS and/or OS.Our study demonstrated that after conducting PSM, patients who received apatinib-based treatment (n = 30) had an ORR of 50.0% and a DCR of 90.0%, compared to 6.7% and 20.0%, respectively, for the chemotherapy-based group (n = 30) (P < 0.001). Notably, statistically significant improvements in ORR and DCR were observed in both the first-line and second-and later-line advanced TNBC populations after receiving apatinib (Table 3). The cohort that was administered apatinib-based treatment exhibited better outcomes than the cohort that received chemotherapy. The PFS and OS periods for the apatinib-based group were 7.8 and 20.3 months, respectively, as opposed to 2.2 and 9.0 months respectively, for the chemotherapy-based group (P < 0.001) (Fig. 2A and C).

The efficacy and safety of the combination group (n = 27) of apatinib and chemotherapy were compared against the chemotherapy alone group (n = 27) using PSM analysis, while considering other phase II trials on TNBC, in order to eliminate any bias created by other therapies. The study showed that there was a substantial improvement in DCR for all populations studied who received a combination of apatinib and chemotherapy, including first-line and second- and later-line advanced TNBC populations. Additionally, there was a noteworthy difference in ORR in the second-line and above populations. However, no substantial difference in ORR was observed in the first-line population (Table 4). The group receiving both apatinib and chemotherapy displayed notable advantages over the group solely receiving chemotherapy in regards to PFS and OS for the entirety of the population. The PFS was found to be 7.8 months in comparison to 2.1 months (P < 0.001) and the OS was 21.1 months in contrast to 9.0 months (P < 0.001) (Fig. 2B and D).

The study showed that the first-line apatinib-based regimen group had a limited sample size and an immature number of PFS and OS events, so statistically significant differences in PFS and OS were not observed for first-line treatments comparing apatinib-based regimens with chemotherapy-based regimens. After excluding treatment factors such as PD1, the sample size of the first-line treatment in the apatinib combined with chemotherapy group was insufficient for statistically significant analyses. Therefore, while there was a tendency towards an ORR benefit in the first-line apatinib combined with chemotherapy group compared to the chemotherapy-only group, no statistically significant difference was found. These limitations are expected to be addressed by conducting prospective, controlled studies on a larger sample size in the future. Despite the small sample size, the study showed that the combination of apatinib and chemotherapy significantly improved ORR, DCR, PFS and OS in comparison to the chemotherapy alone cohort in the overall population. In brief, illustrating that tumor regression was evident, a significant proportion of patients with CBR could ultimately achieve a substantial survival benefit in terms of PFS and OS. This suggests that apatinib combination chemotherapy can facilitate long-term survival through tumor shrinkage or stabilization.

Multivariate analyses revealed that in both cohort1 and cohort2, ECOG score (0 versus 1–2), use of apatinib and clinical benefit profile were independent PFS predictors. However, except for age (less than 50 years versus greater than or equal to 50 years), which was an independent prognostic factor for PFS in cohort1. Additionally, the multivariate analyses indicated that apatinib combination therapy and clinical benefit profile served as independent predictors for prolonged OS in both cohort1 and cohort2.This study suggests that a favourable ECOG score, administration of apatinib, and a positive clinical benefit profile may lead to improved PFS in patients with advanced TNBC. The combined treatment of apatinib and standard therapy may result in a greater decrease in tumour burden and therefore contribute to increased OS. The SHAP values’ summary plots for the leading ten significant predictors of PFS and OS are demonstrated in Fig. 5A and B, correspondingly. The most significant predictor of both PFS and OS was apatinib treatment, followed by age, ECOG PS and other chemotherapy in predicting PFS, and eribulin, other chemotherapy, ECOG PS and age in predicting OS (Fig. 5).

Hypertension, proteinuria, and hand-foot syndrome are the primary side effects of anti-angiogenic medications which can be managed by adjusting the dosage. In the apatinib combination chemotherapy group, the most common grade 3/4 haematological toxic reactions were thrombocytopenia (2.9%) and neutropenia (8.8%). Notably, the incidence of neutropenia was higher in the chemotherapy group (26.8%) than in the apatinib combination chemotherapy group (8.8%) (Table 6). This difference is likely due to the higher proportion of patients in the chemotherapy group receiving Paclitaxel and/or Docetaxel (35.1% in the chemotherapy alone group compared to 5.9% in the apatinib combination chemotherapy group, p = 0.002) (Table 2).The most prevalent non-hematologic toxicities were hand-foot syndrome (35.3%), proteinuria (26.5%), hypertension (61.8%), increased alanine aminotransferase (26.5%), and fatigue (35.3%). In terms of grade 3/4 non-hematologic toxicities, hand-foot syndrome (2.9%) and hypertension (5.9%) were the most commonly observed, nevertheless, fatigue, increased alanine aminotransferase, or proteinuria were not seen(see Tables 5 and 6). According to the phase IIb study of apatinib in TNBC, hand-foot syndrome (17.0%), proteinuria (13.6%), hypertension (11.9%), increased alanine aminotransferase (11.9%), and fatigue (3.4%) were the most frequent grade 3/4 non-hematologic effects^14^. In contrast to the recommended single-agent apatinib dose of500 mg/day, our study shows that low-dose apatinib (250 mg/day) administered alongside chemotherapy significantly reduces grade 3/4 non-hematologic toxicities such as hand-foot syndrome, proteinuria, and hypertension, improving long-term medication compliance. Patients with hypertension should regularly monitor their blood pressure and take prescribed medication. Those with grade 3 or 4 hypertension should cease taking apatinib and adjust their treatment plan based on their blood pressure readings and symptoms. Additionally, if severe hypertension recurs after adjusting the dose of apatinib or prescribing medication for high blood pressure, apatinib use should be discontinued. To alleviate hand-foot syndrome, it may be appropriate to administer vaseline ointment topically and celecoxib for pain management. Temporary cessation of apatinib is necessary along with symptomatic treatment for cases of grade 3/4. Additionally, discontinuation of apatinib is warranted due to the worsening of hand-foot syndrome following dose adjustment and medication use. The study showed that most non-haematological adverse events (AEs) were of mild-to-moderate severity and were manageable with supportive treatment and/or dose modification. Low-dose apatinib enhances long-term drug adherence.

The comparative study shows that even administering low-dose apatinib (250 mg/day) in combination with chemotherapy may be superior to chemotherapy alone, dramatically increasing both short- and long-term efficacy. Additionally, evidence suggests that using the lower dose of apatinib (250 mg/day) in combination with chemotherapy could be more effective than utilizing the higher dose of single-agent apatinib (500 mg/day), while also reducing non-hematologic side effects such as proteinuria, hand-foot syndrome, and hypertension and improving long-term medication adherence. These findings have important implications for the management of metastatic TNBC and require further research to establish their clinical relevance.

Although our study could not stratify the population with a PD-L1 advantage because of the small number of patients receiving PD-1 inhibitors, we aimed to explore whether combining apatinib with a PD-1 inhibitor treatment mode could raise the efficacy. Earlier studies have shown the synergistic mechanism and therapeutic benefits of combining apatinib with a PD-1 inhibitor. Based on previous studies, apatinib has the potential to improve the efficacy of camrelizumab, a PD-1 inhibitor, through a synergistic effect. In a mouse model, apatinib was found to enhance the sensitivity of PD-1 inhibitors by normalising blood vessels, increasing CD8 + T cell and B cell infiltration, and boosting PD-1 expression on immune cells, thus increasing its effectiveness [17]. A phase II study has demonstrated the synergistic impact of apatinib and camrelizumab on advanced TNBC. Patients with metastatic TNBC who had undergone up to the second-line chemotherapy were treated with camrelizumab (200 mg every three weeks) and apatinib (250 mg/day).The study determined an ORR of up to 43.3% and a median PFS of 3.7 months [18]. In a further phase II trial, chemotherapy was combined in a bid to enhance the effect. The approach involved the administration of camrelizumab, a 200 mg dose on day 1, as well as low-dose apatinib, 250 mg daily, while eribulin was given at 1.4 mg/m2 on days 1 and 8 every 21 days. The trial was aimed at 46 previously treated advanced TNBC patients, revealing significant benefits with an ORR of 37.0% and a DCR of 87.0%. Despite the median treatment line being the third line, the median PFS was notably long, amounting to 8.1 months, which was better than the standard treatment typically offered to advanced TNBC patients later in the treatment cycle. The triple combination of camrelizumab, apatinib, and eribulin has been reported to have potential anti-tumor activity in patients resistant to prior immune checkpoint inhibitors (ICIs). This study evaluated 8 patients (17.4%) who had received prior chemotherapy and PD-1/PD-L1 inhibitors, with 2 achieving partial remission (PR) and 5 maintaining stable disease (SD) [19].

Our study’s results revealed the therapeutic advantages of utilizing low-dose apatinib in conjunction with chemotherapy. Notably, our retrospective study used real-world data that may not have undergone the rigor of randomized controlled trials. Furthermore, the lengthy follow-up period, small sample size, and lack of essential clinical criteria such as combination therapy imply that some bias may be present. According to earlier research,pVEGFR2 in breast cancer tissue is reported to be higher compared to normal controls [20], the increased activated protein form of tumor cell pVEGFR2 expression (but not the total VEGFR2),was associated with a significantly improved CBR(81.8 vs. 38.5% among pVEGFR2 higher vs. lower expression patients) and PFS (6.44 vs. 1.97 months).The potential correlation linking greater expression of pVEGFR2 in tumour tissue with apatinib efficacy is significant and requires urgent confirmation in forthcoming studies of TNBC treated with apatinib or similar pVEGFR2-targeting TKI [14]. Unfortunately, we were unable to obtain tumor samples from other hospitals, so we cannot determine the difference in overall pVEGFR2 between the apatinib and non-apatinib groups. Nevertheless, other research shows that apatinib enhances the anti-tumor effect of PTX on TNBC cells through the molecular pathway of PI3K/p65/Bcl-xl. This combination of apatinib and microtubule inhibitor shows promise in the treatment of TNBC [21]. In our research, after PSM, almost 40% of patients in the apatinib group received microtubule inhibitors, and the percentage of patients who received microtubule inhibitors (including Paclitaxel/Docetaxel, nab-paclitaxel, eribulin) was higher than that of the chemotherapy alone group, which may contribute to the higher efficacy observed in the apatinib group. We are presently undertaking an exploratory clinical study of apatinib mesylate, in combination with nab-paclitaxel, for the second-line treatment of advanced triple-negative breast cancer. This study is investigator-initiated and has been approved by both the Ethics Committee of Fujian Cancer Hospital (ethic code: K2021-122-01) and the Chinese Ethics Committee of Registering Clinical Trials (ethic code: CHiECRCT20210338). We will evaluate the effectiveness of combining apatinib and nab-paclitaxel in the treatment of triple-negative breast cancer tumors transplanted in mice. The objective is to reinforce the effectiveness of apatinib in combination with chemotherapy for salvage therapy in triple-negative breast cancer and investigate the correlation between pVEGFR2 expression in tumor tissue and response prediction. Therefore, to comprehensively examine the potential advantages of this approach, forthcoming clinical randomized controlled trials should strive to amplify the sample size whilst considering other parameters including pVEGFR2.

## Conclusion

Our research reveals that combining low-dose apatinib (250 mg per day) with chemotherapy yields a superior Disease Control Rate (DCR) benefit compared to chemotherapy alone, accompanied by favorable Progression-Free Survival (PFS) and Overall Survival (OS) advantages. Moreover, the administration of low-dose apatinib significantly reduces the incidence of hypertension, proteinuria, and hand-foot syndrome. The majority of non-hematological adverse events are of mild to moderate severity and can be managed through supportive measures or dosage adjustments. Notably, this reduced apatinib dosage demonstrates increased treatment adherence compared to the previously recommended 500 mg/day dosage in prior studies. Consequently, a lower dose of apatinib (250 mg/day) combined with chemotherapy may be as effective as the recommended 500 mg/day dose, warranting further exploration of this combined treatment approach. Additionally, it is crucial to investigate which chemotherapeutic agents exhibit superior synergistic effects when used with apatinib and to verify whether tumor pVEGFR2 expression can serve as an efficacy predictor for apatinib chemotherapy.

## Data Availability

The datasets used and/or analyzed in the study are available from the corresponding author on reasonable request.
