# Correction: A real-world study of the effectiveness and safety of apatinib-based regimens in metastatic triple-negative breast cancer

**DOI:** 10.1186/s12885-024-12150-8

**Published:** 2024-03-25

**Authors:** Weiwei Huang, Chenxi Wang, Yangkun Shen, Qi Chen, Zhijian Huang, Jian Liu, Xiaoyan Lin, Lili Wang, Fan Wu, Xinhua Chen, Nani Li, Yi Hong, Mulan Chen, Jieyu Li, Chuanzhong Huang

**Affiliations:** 1 Department of Medical Oncology, Fujian Medical University Union Hospital, No.29, Xinquan Road, Gulou District, 350001 Fuzhou, Fujian province China; 2 Department of Medical Oncology, Clinical Oncology School of Fujian Medical University, Fujian Cancer Hospital, No.91, Fuma Road, Jin’an District, 350014 Fuzhou, Fujian province China; 3 Fujian Key Laboratory of Translational Cancer Medicine, Fujian Cancer Hospotial, No.91, Fuma Road, Jin’an District, 350014 Fuzhou, Fujian province China; 4 Fujian Key Laboratory of Innate Immune Biology, Biomedical Research Center of South China, Fujian Normal University Qishan Campus, 350117 College Town, Fuzhou, Fujian Province PR China; 5 Department of Breast Surgical Oncology, Clinical Oncology School of Fujian Medical University, Fujian Cancer Hospital, No. 91, Fuma Road, Jin’an District, 350014 Fuzhou, Fujian province China; 6 Laboratory of Immuno-Oncology, Clinical Oncology School of Fujian Medical University, Fujian Cancer Hospital, No.91, Fuma Road, Jin’an District, 350014 Fuzhou, Fujian province China

**Correction: BMC Cancer 24, 39 (2024)**

10.1186/s12885-023-11790-6

Following publication of the original article [1], a typesetting error was noticed in the equal contributions statement. The equal contributions should read as follows: † Weiwei Huang and Chenxi Wang contributed equally to this work.

The original article [1] has been corrected.
